# Susceptibility of Different Hepatitis B Virus Isolates to Interferon-Alpha in a Mouse Model Based on Hydrodynamic Injection

**DOI:** 10.1371/journal.pone.0090977

**Published:** 2014-03-11

**Authors:** Jingjiao Song, Yun Zhou, Sheng Li, Baoju Wang, Xin Zheng, Jun Wu, Kathrin Gibbert, Ulf Dittmer, Mengji Lu, Dongliang Yang

**Affiliations:** 1 Division of Clinical Immunology, Tongji Hospital, Tongji Medical College, Huazhong University of Science and Technology, Wuhan, P.R. China; 2 Experimental Medicine Center, Tongji Hospital, Tongji Medical College, Huazhong University of Science and Technology, Wuhan, P.R. China; 3 Department of Infectious Diseases, Union Hospital of Tongji Medical College, Huazhong University of Science and Technology, Wuhan, P.R. China; 4 Institute of Virology, University Hospital of Essen, University of Duisburg-Essen, Essen, Germany; Yonsei University, Republic Of Korea

## Abstract

Interferon alpha (IFN-α) is commonly used for the treatment of chronic hepatitis B (CHB) patients. Many factors including viral genetics may determine the outcome of IFN-α therapy. In this study, we tested whether the expression of IFN-α directly in the liver inhibits HBV gene expression and replication using a HBV hydrodynamic injection (HI) mouse model. Two replication-competent clones from different HBV isolates that belonging to HBV genotype A and B based on a pAAV vector (pAAV-HBV-A and pAAV-HBV-B) were compared for their susceptibility to IFN-α. HBV clones were injected into mice either alone or in combination with a murine (m) IFN-α expression plasmid (pmIFN-α). HBsAg and HBeAg concentrations and HBV DNA levels in mice differed after injection of these two HBV clones. Co-application of pmIFN-α together with the two distinct isolates resulted in markedly different kinetics of decline of HBsAg, HBeAg, and HBV DNA levels in the mice. Immunohistochemical staining of liver sections with anti-HBc showed that mIFN-α application completely inhibited the expression of HBcAg in mice inoculated with pAAV-HBV-B, whereas the expression of HBcAg was only reduced in mice with pAAV-HBV-A. Consistently, mice injected with pAAV-HBV-B and pmIFN-α showed higher expression levels of the IFN-stimulated genes (ISGs) ISG15, OAS, PKR as well as proinflammatory cytokine IL-6 in the liver. In addition, expression levels of anti-inflammatory cytokine IL-10 was down-regulated significantly in liver of the mice injected with pAAV-HBV-B and pmIFN-α. Our data demonstrate that IFN-α exerts antiviral activity in HBV mouse model, but different HBV isolates may have diverse susceptibility to IFN-α.

## Introduction

Hepatitis B virus (HBV) is a DNA virus causing chronic infections of the human liver [1]. More than 400 million people worldwide have been infected and about one million patients die annually from HBV infection [2]. The genetic analysis of HBV has revealed the existence of ten different genotypes (A–J) [3]. In Asia, HBV infection is highly prevalent and HBV isolates commonly belong to genotypes B and C.

Currently, inhibition of HBV infection can be achieved by suppressing viral replication. IFN-alpha (IFN-α) and nucleoside/nucleotide analogs [4]–[6] are mainly used for therapies of chronic hepatitis B (CHB). In contrast to nucleoside analogs, IFN-α is able to enhance host immune responses and promote cellular immune responses, mediating inhibition of HBV replication and gene expression [7]–[8]. IFN-α is an approved and effective treatment for CHB, inducing HBeAg seroconversion and histological remission in about one third of the patients [9]. In addition, several previous studies have suggested that IFN-α therapy may have long-term beneficial effects in terms of viral clearance, prevention of HCC and prolonging survival in patients with CHB [10]–[11]. Compared to nucleoside/nucleotide analogs, the advantage of IFN-α therapy includes a more durable response, with the benefit of lacking emergence of drug resistant HBV mutants during the course of treatment. Further, HBsAg loss associated with standard IFN-α therapy has been reported in European studies, with the loss of HBsAg within 1 year of treatment in 5-10% of recipients [12].

However, only 30% of the CHB patients showed sustained response to IFN-α treatment, which limits the clinical effect and application of IFN-α [13]. The mechanisms underlying the low response to IFN-α in CHB patients remain unclear. It has been considered that it involves multiple causes including both virus-related and host-specific factors [14]–[16]. Previous studies showed that the HBV genotype has little impact on the outcome of CHB patients treated with nucleoside/nucleotide analogues [17]–[18]. However, different genotypes of HBV showed distinct response to IFN-α treatment [19]–[20]. Different genotypes of HBV may have distinct virologic characteristics, which may correlate with antiviral curative effects and clinical outcome [21]–[22]. It has been suggested that IFN-α-based therapies have higher efficacy in patients including HBeAg-positive and HBeAg-negative patients with genotype A versus D, and in HBeAg-positive patients with genotype B versus C [19]. However, it was not yet demonstrated that whether the different HBV isolates display various susceptibility to IFN-α, considering that virus genetic alteration during antiviral therapy is an important factor that influenced the antiviral effect.

The type I IFNs belong to a multigene family with over 14 IFN-α subtypes in humans [23] and over 10 IFN-α subtypes in mice [24]. There is 80–95% homology at the amino acid level among various IFN-α subtypes [25]. The IFN-α mediated complex antiviral responses include immune regulation and the induction of cytokines and antiviral proteins. The IFN-α signaling is mediated by the JAK-STAT pathway and leads to the activation of a large number of interferon-stimulating genes (ISGs) [26]–[28]. These effectors individually may block viral transcription, degrade viral RNA, inhibit translation or modify protein function to inhibit the process of viral replication.

The antiviral activity of IFN-α against HBV could be demonstrated *in vitro* and in an HBV transgenic mouse model [29]–[31]. It has shown that IFN-α may destabilize HBV nucleocapsids at a post-transcriptional step [32]. Tian et al. have shown that IFN-α may exert complex antiviral activities in different HBV transgenic mouse strains, depending on the replication status of HBV [33]. Zhang et al. reported that the effectiveness of Peg-IFN-α to suppress HBV replication is dependent on different HBV genotypes in HBV HI mouse model by subcutaneously injection of Peg-IFN-α [34]. However, it was not yet demonstrated whether directly application of IFN-α in the mouse liver displays various antiviral effect between HBV isolates and it was not yet possible to compare genetically different HBV isolates for their susceptibility to IFN-α. In this paper, the antiviral effect of mIFN-α against two different HBV isolates belonging to the genotypes A and B was determined in a hydrodynamic injection (HI) mouse model.

It is worth mentioning that injection of recombinant IFN-α distributes IFN-α in the organism and only a part of IFN-α could reach the liver. It is clear that IFN-α is diluted and the major part will be metabolized in the body. Thus, we attempted to test the application by HI where the plasmids could be delivered into the liver and express IFN-α protein there. In addition, eukaryotic proteins have some post-translational modifications which are required for its biological activities. The hepatocytes will produce fully functional IFN-α with complete post-translational modifications. Therefore, the expression of IFN-α in the liver by HI method may achieve a better therapeutic effect for the treatment of chronic HBV infection.

For this experiment, we chose IFN-α4, as this subtype has similar properties like IFN-β and is able to induce the expression of other subtypes [35]. Co-application of pmIFN-α together with the HBV clones led to rapid but kinetically different decline of HBsAg, HBeAg, and HBV DNA levels in sera of the mice. Interestingly, 3 ISGs ISG15, OAS, PKR and proinflammatory cytokine IL-6 were up-regulated, anti-inflammatory cytokine IL-10 was down-regulated in liver tissue after pmIFN-α injection, but to different degrees for the two HBV isolates.

### Ethics Statement

The animal experiments were carried out in concordance with the guidelines and protocols established by the Institutional Animal Care and Use Committee (IACUC Number: 287) at the Huazhong University of Science and Technique and the Tongji Hospital of Tongji Medical College. IACUC at the Huazhong University of Science and Technique and the Tongji Hospital of Tongji Medical College approved this study. All surgeries were performed under sodium pentobarbital anesthesia, and every effort was made to minimize suffering.

## Materials and Methods

### HBV Genotype B infectious clone and pmIFN-α expression plasmid

A *Not*I digested and blunted fragment of 1.3 fold genotype B HBV DNA including nt 1038-3215-1984 (GenBank accession number AY220698.1) from pAAV-HBV1.3 [36] was cloned into the *Sac*II/*Bgl*II digested and blunted pAAV vector derived from pAAV-HBV1.2 [37] (kindly provided by Prof. Pei-Jer Chen, National Taiwan University). The resulting plasmid named pAAV-HBV-B contains the HBV fragment spanning nucleotides 1038-3215-1984 flanked by inverted terminal repeats of AAV.

In this paper, pAAV-HBV1.2 was renamed as pAAV-HBV-A to distinguish from pAAV-HBV-B. The detail of construction procedure was shown in supplementary material. The expression plasmid pkCMVint-mIFN-α4 (pmIFN-α) contained the full length murine *IFN-α4* gene [38].

### Preparation of HI mouse model

Adult SPF C57BL/6 mice (male, 6–8 weeks old) were purchased from the breeding colonies of the experimental animal center in Shanghai, China, and maintained in a 12-hour light-dark cycle, and cared for in accordance with national and local regulations. HI experiments were carried out as described previously [37]. 10 µg pAAV-HBV-A or pAAV-HBV-B together with 20 µg pmIFN-α were co-injected into the tail vein of mice in a volume of 0.9% NaCl equivalent to 8% of the mouse body weight. The total volume was delivered within 5–8 s. The mice injected only with pAAV empty vector or 0.9% NaCl were used as negative control and the mice injected only with pAAV-HBV-A, pAAV-HBV-B or pmIFN-α, separately, were used as positive control. Each group included at least 10 mice.

### Detection of mIFN-α protein in mouse serum samples

The amount of mIFN-α protein in mouse serum samples were collected at 24 hour post injection (hpi), 1, 10, 20, 30, 50 day post injection (dpi) and determined by commercial enzyme-linked immunosorbent assay (ELISA) kit (BD Biosciences, New Jersey, USA). The serial dilutions of mIFN-α standard samples from 12.5 to 500 pg/ml were used to establish the standard curve. The sera of mice used in this test were 2-fold diluted. The mIFN-α standard samples were measured in duplicates.

### Detection of HBsAg and HBeAg in mouse sera

The serum specimens were collected and assayed for HBsAg and HBeAg at 1, 4, 7, 10, 15, 20, 25, 30, 40, 50 and 60 dpi. HBsAg and HBeAg were determined by using commercial enzyme-linked immunosorbent assay (ELISA) kits (Kehua, Shanghai, China). 10-fold diluted serum samples were used for detection.

### Isolation and analysis of HBV DNA in mouse sera

The serum HBV DNA were detected at 1, 4, 7, 10, 15, 20, 25, 30, 40, 50 and 60 dpi.

HBV DNA was extracted from 10 µl mouse serum sample using the following method : 10 µl mouse serum was added into 40 µl PBS, and digested by 10 µg DNaseI for 1 hr at 37°C, then 100 µl lysis buffer (20 mM Tris-HCl, 20 mM EDTA, 50 mM NaCl, and 0.5% SDS) containing 50 µg proteinase K was added; after incubation at 65°C overnight, viral DNA was isolated by phenol/chloroform extraction and ethanol precipitation. The DNA pellet was rinsed with 70% ethanol and resuspended in 10 µl ddH_2_O.

The quantification of HBV DNA was performed by using a routine real time PCR procedure described previously [39]-[43]. The HBV DNA copy numbers were determined by SYBR Green Real time PCR Master Mix (commercially available assay kit, TOYOBO, Osaka, Japan). Melt curve analysis and agarose gel electrophoresis were used to verify the specificity of the real-time PCR. The following primers were used: forward primer: 5′-CTG CAT CCT GCT GCT ATG-3′ (nt 408-425), and reverse primer: 5′-CAC TGA ACA AAT GGC AC-3′ (nt 685-701) according to the reference sequence with Genbank accession number (AY220698.1). A serum sample containing a known concentration of HBV DNA was used as positive control.

### Immunohistochemistry

Liver tissue was taken from the mice receiving HI at 1, 10, 20, 30 and 50 dpi and embedded in paraffin. Intrahepatic HBcAg expression was visualized by immunohistochemical staining of tissue sections by rabbit anti-HBc (Dako). The liver sections were also stained with hematoxylin. Staining was repeated three times for each sample.

### Purification of RNA from mouse liver tissue and Real-Time PCR detection

Total RNA was isolated from collected liver tissue at indicated time points by commercial kits (OMEGA, Norcross, USA). RNA was reverse-transcribed and the product was used for analyzing the copy number of mouse ISG15, OAS, PKR, IL-6, IL-10 and TGF-β cDNA by using real-time quantitative PCR (RT-PCR). Primers for real-time detection are ordered from Qiagen Company (California, USA).

### Enzyme-linked immunospot (ELISPOT) assay

ELISPOT assay was carried out using the mIFN-γ precoated ELISPOT Kit (Dakewe, Shenzhen, China) according to the manufacturer's instructions. Briefly, 96-well flat-bottomed microtiter plates were preincubated with the coating antibody (anti-mIFN-γ monoclonal antibody) at 4°C overnight and blocked for 2 hr at 37°C. Mouse splenocytes were added to wells at the density of 2×10^5^ cells per well in triplicate with 0.5 µg/ml of full length recombinant HBsAg and HBcAg peptide separately and incubated at 37°C, 5% CO_2_ for 24 hr. 5 µg/ml of ConA (Sigma, St. Louis, USA) was used as positive control and 0.5 µg/ml of CMV (prospect) as negative control. Thereafter, cells were removed. Wells were washed ten times with PBS containing 0.05% Tween-20 (PBS-T) and incubated with 100 µl of biotinylated anti-IFN-γ antibody for 1 hr. The plates were washed again with PBS-T and incubated with 50 µl HRP-strepto-avidin solution at 37°C for 1 hr. Spot-forming cells were counted and analyzed with an ELISPOT plate reader (BioReader 4000, Biosys, Germany). Results were presented as spot-forming cells per 2×10^5^ cells.

### Statistics

Experimental data were reported as means±standard deviations. The *t*-test was applied to comparisons between different groups; a *P* value <0.05 was considered statistically significant. Statistical analysis was performed using SPSS (SPSS, Inc., Chicago, IL).

## Results

### Identification of pAAV-HBV-B

To compare the susceptibility of different HBV isolates to IFN-α in the HI mouse model, a new replication-competent clone belonging to HBV genotype B named pAAV-HBV-B containing 1.3 fold genome of HBV genotype B was constructed. The construction procedure and identification results were shown in Fig. S1A and B separately. We confirmed that the pAAV-HBV-B was constructed correctly.

### Expression of mIFN-α in mouse sera after HI

**Figure 1:**
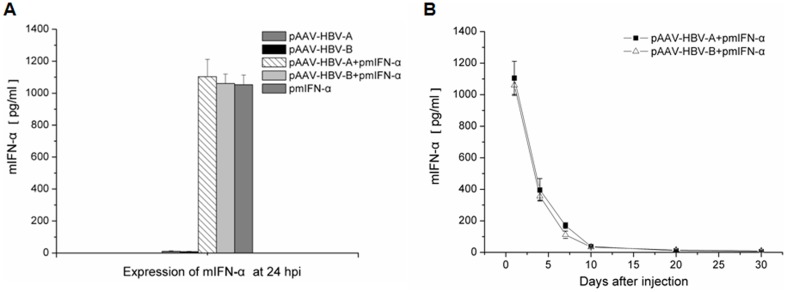
Detection of mIFN-α in serum samples from the mice after HI. Mice received HI with 10 µg of either HBV clones or pmIFN-α alone or combination of HBV clones with pmIFN-α. Mouse sera were collected at the indicated time points. The concentrations of mIFN-α protein were detected by mIFN-α ELISA kit. (A) Expression of mIFN-α in mice at 24 hpi. (B) Kinetics of mIFN-α expression in mice after HI. At least three serum specimens of the mice *per* group were analysed at the indicated time points.

The presence of mIFN-α in sera of mice after HI of pmIFN-α was measured with commercial ELISA kit. Fig. 1A shows that the expression level of mIFN-α at 24 hpi after HI was above 1000 pg/ml, indicating effective expression of mIFN-α in mice after HI of pmIFN-α. We also determined the expression of mIFN-α in the mice over time up to 30 dpi (Fig. 1B). The result has shown that the concentrations of mIFN-α in mice declined with time but remained to be detectable more than 10 days. It is worth pointing out that the peak mIFN-α concentration and the decline was identical if pAAV-HBV-A and pAAV-HBV-B infection clones were co-applied.

### Kinetics of HBsAg, HBeAg and HBV DNA in sera of the mice after HI of HBV clones and pmIFN-α

**Figure 2:**
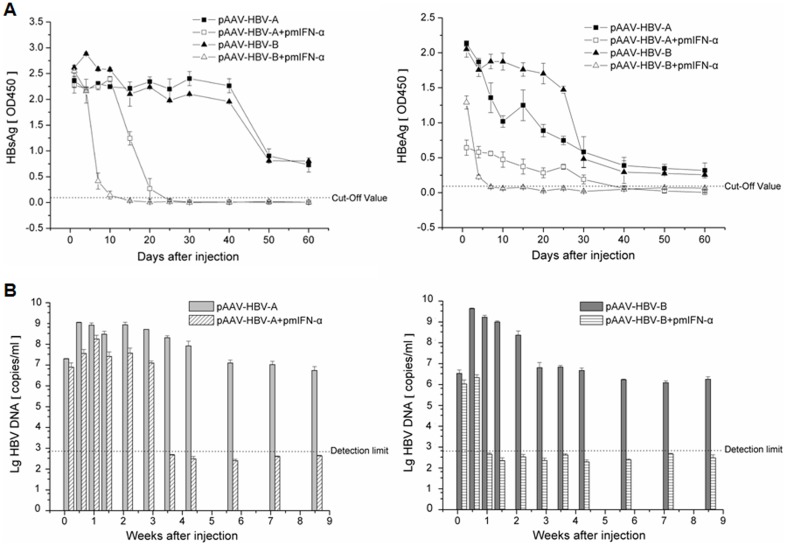
Kinetics of HBsAg, HBeAg and HBV DNA in sera of the mice after HI. 10 µg of pAAV-HBV-B alone or combined with 20 µg pmIFN-α were used for HI. (A) HBsAg and HBeAg levels in the sera of C57BL/6 mice after HI. The value of OD450 ≥ Cut-Off value means the sample was positive. (B) Real-time PCR detection of HBV DNA levels in the sera of the mice after HI, dotted line indicates detection limit. At least three mice *per* group were analysed.

To compare the inhibitory effects of mIFN-α on the replication of different HBV isolates in mice, the kinetics of serum HBsAg, HBeAg and HBV DNA in mice were determined after HI. As shown in Fig. 2, the mice injected with pAAV-HBV-B had the higher HBsAg and HBeAg expression levels during the first two weeks than that with pAAV-HBV-A. However, the serum HBsAg level in the mice injected with pAAV-HBV-B and pmIFN-α significantly declined from 4 dpi on compared to the control group injected with pAAV-HBV-B alone. On 15 dpi, the HBsAg was cleared in all mice of this group. In contrast, serum HBsAg in the mice injected with pAAV-HBV-A and pmIFN-α began to decline at 15 dpi, compared with the control mice injected with pAAV-HBV-A only (Fig. 2A). HBsAg clearance occurred later at day 25 dpi. The HBeAg level decreased from 1 dpi by injecting either pAAV-HBV-B combined with pmIFN-α or pAAV-HBV-A together with pmIFN-α, whereas the HBeAg in the pAAV-HBV-B and pmIFN-α co-injected mice decreased markedly compared with the pAAV-HBV-A and pmIFN-α co-injected mice (Fig. 2A).

Real-time PCR analysis showed that the peak HBV DNA level in sera from mice injected with pAAV-HBV-B was 1 order higher than that of pAAV-HBV-A (Fig. 2B). After co-injection with pmIFN-α, the serum HBV DNA levels declined in both pAAV-HBV-B and pAAV-HBV-A injected mice. Interestingly, after pmIFN-α treatment, HBV DNA in the pAAV-HBV-B injected mice became undetectable after 7 dpi, while HBV DNA was cleared after 25 dpi in the pAAV-HBV-A injected mice (Fig. 2B). Our results suggest that pAAV-HBV-B had higher replication competence than pAAV-HBV-A and led to higher HBsAg and HBeAg expression and HBV DNA levels in mouse sera. Further, the antiviral effect of mIFN-α was more pronounced against pAAV-HBV-B than pAAV-HBV-A, confirming the presumption that different HBV isolates may have different susceptibilities to IFN-α.

### Effect of mIFN-α on the HBcAg expression in liver of the mice injected with HBV clones and pmIFN-α

**Figure 3:**
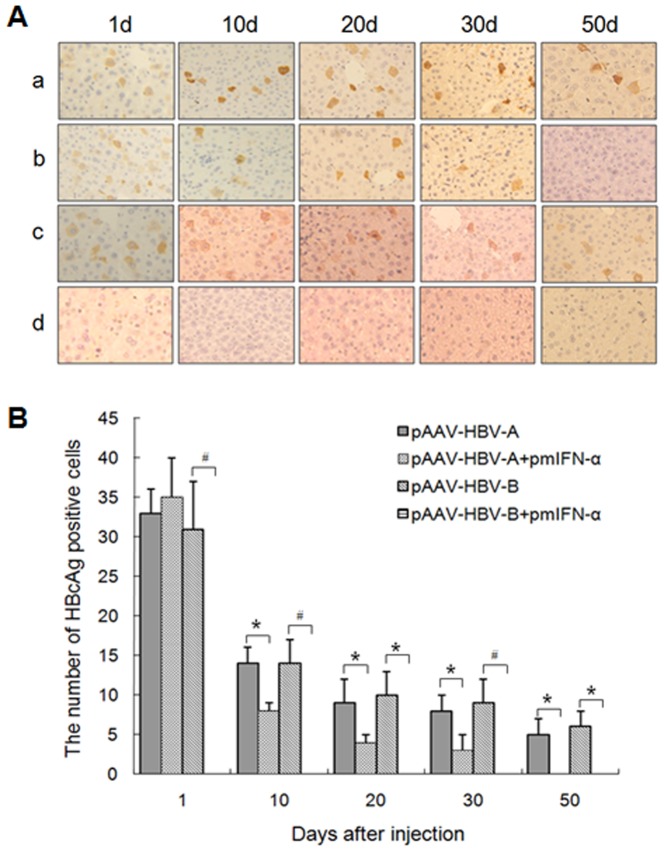
Application of mIFN-α expression vector reduced HBcAg expression in liver of the mice. The mice injected with 10 µg of HBV clones alone or combined wiht 20 µg of pmIFN-α. (A) Immunohistochemical staining of the liver sections for HBcAg in hepatocytes of pAAV-HBV-A- (a), pAAV-HBV-A combined with pmIFN-α- (b), pAAV-HBV-B- (c) and pAAV-HBV-B together with pmIFN-α (d)-injected mice at 1, 10, 20, 30 and 50 dpi (Original magnification: 400X). The mice injected with 0.9% NaCl were used as negative control. Liver specimens of at least three mice *per* group (n≥3) were analyzed. Each sample was done in duplicate. (B) Frequencies of HBcAg positive cells in the mouse liver sections. The data were analyzed by *t* test, and the differences were statistically significant (* means p<0.05 and # means p<0.01).

Immunohistochemical staining was performed to determine the expression of HBcAg in the liver sections of the mice at 1, 10, 20, 30 and 50 dpi. As shown in Fig.3, the control mice injected with either pAAV-HBV-A or pAAV-HBV-B presented comparable HBcAg expression levels in the liver. The number of HBcAg positive cells was only decreased in liver of the mice injected with pAAV-HBV-A and pmIFN-α after 10 dpi whereas HBcAg positive cells were undetectable in the liver of mice injected with pAAV-HBV-B and pmIFN-α during the whole observation period (Fig. 3A and B)

These data indicate that mIFN-α could completely inhibit the expression of HBcAg in liver tissue of the mice injected with pAAV-HBV-B but only partly inhibited the expression of HBcAg in liver of the mice injected with pAAV-HBV-A. Overall, the data of the HBcAg expression in the liver of different mouse groups were well consistent with the data of HBsAg, HBeAg, and HBV DNA detection in mouse sera.

### Induction of ISGs ISG15, OAS and PKR in liver tissue of the mice after application of pmIFN-α

**Figure 4:**
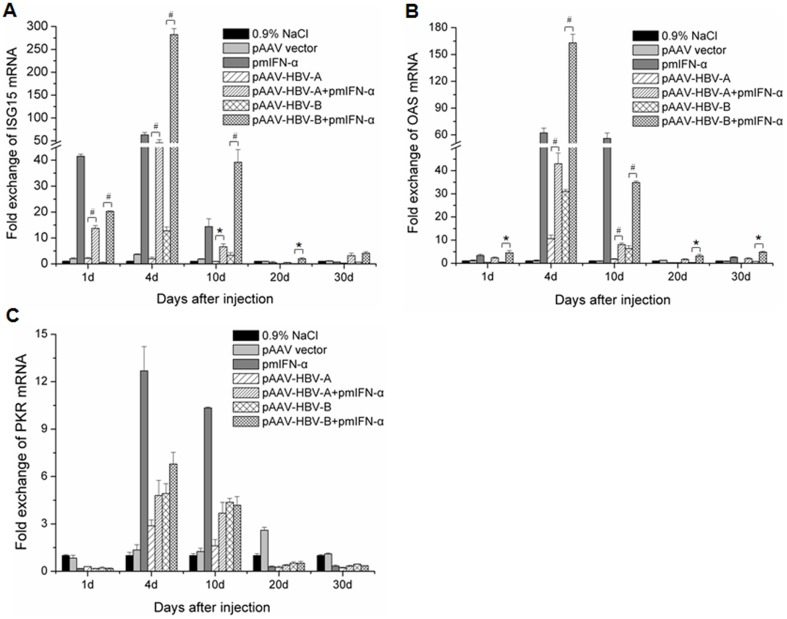
Induction of ISG15, OAS and PKR expression in the mouse liver after HI. Mice received HI with 10 µg of HBV clones or pmIFN-α alone or combination of HBV clones and pmIFN-α. The mice injected with 0.9% NaCl or pAAV vector, were used as control. Total RNA was extracted form liver tissue of the mice at the indicated time points after HI and the levels of ISG (A), OAS (B) and PKR (C) mRNA were determined by quantitative real-time RT PCR. The β-actin mRNAs were quantified for normalization. Each sample was run in duplicate and at least three mouse liver specimens *per* group were analyzed (n≥3). Differences between the groups were analyzed by using the *t* test: * means p<0.05 and # means p<0.01.

To analyze the induction of ISGs expression by application of pmIFN-α and its association with the inhibition of HBV replication in mice, the total RNAs were extracted from the liver tissue of mice at the indicated time points after HI and subjected to real-time RT-PCR for the quantification of the three ISGs ISG15, OAS and PKR. The copy numbers of ISG mRNAs were normalized in contrast to those of beta-actin. As shown in Fig. 4, the application of pmIFN-α led to a significant up-regulation of the mRNA levels of all 3 ISGs but with significantly different kinetics. While ISG15 and OAS were already induced by the application of pmIFN-α at 1 dpi and had the peak expression levels at 4 dpi in the liver of mice. The PKR mRNA level was low at 1 dpi and up-regulated only weakly at 4 dpi and 10 dpi. When co-applied with HBV clones, pmIFN-α induced ISGs expression in the liver of mice (Fig. 4). Though mIFN-α was produced in mice at comparable levels with and without HBV clones (Fig. 1), the kinetics of ISGs induction was changed in the presence of HBV lones. First, the induction of ISG15 (Fig. 4A), OAS (Fig. 4B) and PKR (Fig. 4C) by pmIFN-α were generally reduced in mice with HBV clones compared with that in mice with pmIFN-α alone. The ISGs expression in mice injected with pAAV-HBV-A and pmIFN-α remained lower at 4 dpi and 10 dpi compared with the control mice injected with pmIFN-α alone, consistent with the fact that HBV continued to replicate at this time point. In contrast, ISGs expression in the liver of the mice receiving pAAV-HBV-B was strongly up-regulated at 4 dpi, associated with the early HBV clearance at 7 dpi. Taken together, HBV might inhibit the induction of ISGs by mIFN-α.

### Detection of IL-6, IL-10 and TGF-β in liver tissue of the mice after application of pmIFN-α

**Figure 5:**
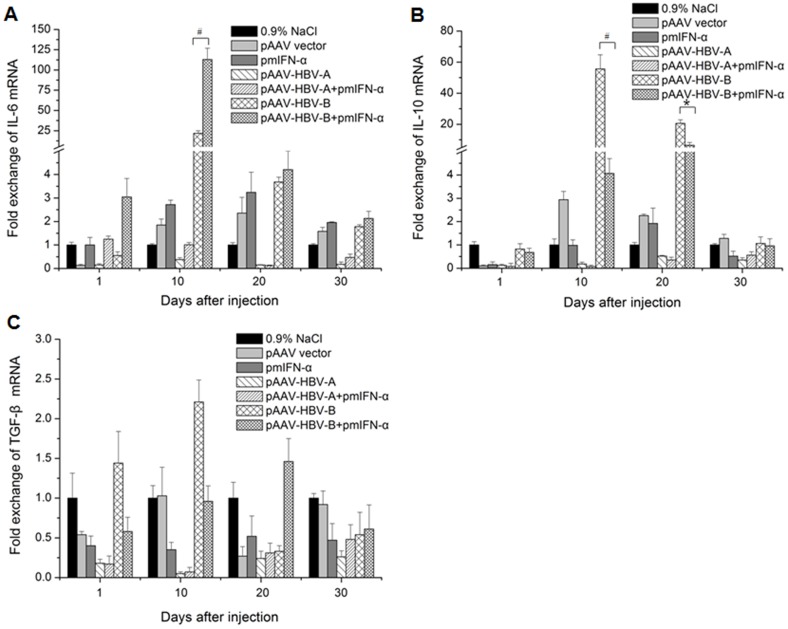
Expression levels of IL-6, IL-10 and TGF-β in the mouse liver after HI. Mice received HI with 10 µg of HBV clones or pmIFN-α alone or combination of HBV clones and pmIFN-α. The mice injected with 0.9% NaCl or pAAV vector, were used as control. Total RNA was extracted form liver tissue of the mice at the indicated time points after HI and the levels of IL-6 (A), IL-10 (B) and TGF-β (C) mRNA were determined by quantitative real-time RT PCR. The β-actin mRNAs were quantified for normalization. Each sample was run in duplicate and at least three mouse liver specimens *per* group were analyzed (n≥3). Differences between the groups were analyzed by using the *t* test: * means p<0.05 and # means p<0.01.

To know the expression levels of proinflammatory and anti-inflammatory cytokine in mice liver were influenced by injection of pmIFN-α. mRNA levels of IL-6, IL-10 and TGF-β were detected by RT PCR. The copy numbers of IL-6, IL-10 and TGF-β mRNAs were normalized against those of beta-actin. As shown in Fig. 5, the application of pmIFN-α led to an up-regulation of the IL-6 and down-regulation of IL-10 mRNA levels from 1 day after injection. By comparison, IL-6 expression was strongly up-regulated (Fig. 5A) and IL-10 expression was strongly down-regulated (Fig. 5B) in the liver of the mice receiving pAAV-HBV-B at 10 dpi. However, the mRNA levels of TGF-β in liver of the mice has no significant variation between those two groups (Fig. 5C).

### HBV-specific CD8^+^ T cells responses in spleen of the mice after HI

**Figure 6:**
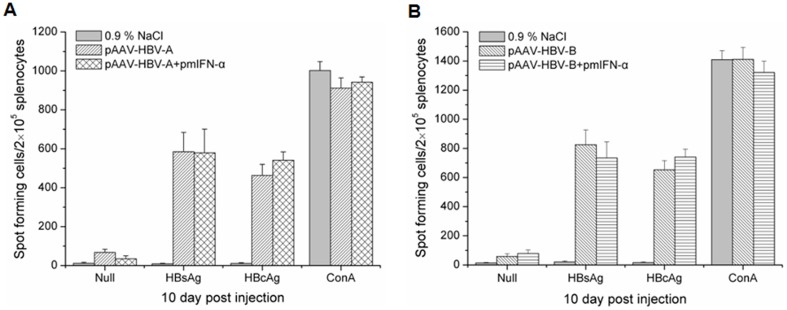
Detection of T-cell responses to HBsAg and HBcAg in mice received HI with HBV clones and pmIFN-α. The specific T-cell responses to the full length HBsAg and HBcAg in mice were analyzed by ELISPOT assays after HI with HBV clones and pmIFN-α. The numbers of HBsAg and HBcAg specific IFNγ secreting cells in 2×10^5^ splenocytes of the mice HI with pAAV-HBV-A and pmIFN-α (A) or HI with pAAV-HBV-B and pmIFN-α (B) at 10 d by IFNγ ELISPOT assay in the presence of 0.5 µg/ml full length HBsAg or HBcAg peptide. 5 µg/ml of ConA was used as positive control and 0.5 µg/ml of CMV was used as negative control (data not shown). The experiments were done in duplicate and spleen specimens of at least three mice *per* group were analyzed (n≥3). The data were analyzed by *t* test but no significant difference between the experimental groups was found (p>0.05).

IFN-α may enhance HBV-specific adaptive responses. To investigate the function of specific T-cells, HBV-specific T cell responses were detected at 10 dpi by ELISPOT assay detecting mIFN-γ producing cells. In both control and pmIFN-α treated mice, the mIFN-γ producing cells stimulated with recombinant HBcAg and HBsAg were detectable at 10 dpi (Fig. 6). There was no significant difference in the numbers of mIFN-γ producing cells in mice receiving HBV clones alone and with pmIFN-α together. Apparently, HBV-specific T cell responses were not promoted by mIFN-α within 10 dpi and therefore not primarily responsible for the rapid clearance of HBV from mice, as in mice injected with pAAV-HBV-B (Fig. 6B).

## Discussion

In the present study, we showed that HI with mIFN-α expression plasmid could induce IFN-α production in mice, trigger the ISGs expression in the mouse liver, and effectively suppressed HBV replication in HBV HI mouse model. Our major finding in the present study is that genetically distinct HBV isolates may have significantly different susceptibility to mIFN-α *in vivo*. The methodology established in this study will be useful to dissect the viral determinants for IFN-α susceptibility.

The production of mIFN-α in mice was detected over a period of more than 10 dpi. This finding is useful to achieve prolonged IFN-α expression *in vivo* for mechanistic studies. Previously, polyinosinic-polycytidylic acid (polyIC) was used to induce IFN-α/β expression to influence HBV replication [33], [44]. In our experience, poly IC induced only a short term expression of IFN-α/β and was not active after 4 dpi (Wu et al., unpublished results). Thus, HI application of pmIFN-α plasmid triggers a kinetically different mIFN-α expression than polyIC and led to long lasting upregulation of ISGs at least up to 30 days in some mice (Fig. 4). The peak levels of ISGs expression were detected at 4 dpi, though the highest level of mIFN-α production was measured at 1 dpi. Thus, the continuous mIFN-α stimulation over days may be important to achieve a high level expression of ISGs and HBV clearance. In addition, to know whether application of pmIFN-α influnced the inflammatory reaction in liver of the mice, mRNA levels of IL-6, IL10 and TGF-β were detected. We found that proinflammatory cytokine IL-6 was up-regulated and anti-inflammatory cytokine IL-10 was down-regulated significantly at 10 dpi in liver tissue of the mice co-injected with HBV and pmIFN-α compared with the mice injected only with HBVclones.

HI of pmIFN-α was effective to clear HBV from mice. The suppressive effect of mIFN-α on HBV was already pronounced at 1 dpi. Though HBsAg expression was not significantly inhibited at 1 dpi, serum HBeAg and HBV DNA levels were strongly reduced. Dependent on HBV isolates, HBV markers were completely cleared on 7 or 25 dpi. Consistent with previous publications about HBV transgenic mice, HBV is highly sensitive to the antiviral activity of mIFN-α. However, HBV replication rebounded in HBV transgenic mice as the antiviral activity of mIFNs ceased [30], [32]. Here, we could show that HBV could be completely eliminated if the HBV genome is not integrated in the host genome. Strikingly, HBcAg detection by immunostaining was strongly reduced at the very beginning in mice with pAAV-HBV-B which appeared to be very sensitive to mIFN-α. This finding is consistent with the assumption that IFN-α may prevent the formation of HBV nucleocapsid. We also detected HBV-specific CD8^+^ T cells responses in the spleen of the mice after HI. HBV-specific T cell responses were not promoted by the application of mIFN-α plasmids within 10 dpi and therefore not primarily responsible for the rapid clearance of HBV in these mice. Moreover, we also detected mRNA levels of mIFN-γ in the liver of mice at different time points and found there were no significant difference between the HBV injected group and mIFN-α treated group (data not shown). In addition, we performed HI with pAAV-HBV and injected the mIFN-α plasmids 14 days later. The results obtained in this experiment showed that HBsAg, HBeAg and HBV DNA levels in mice declined after the injection of the mIFN-α plasmids with kinetics similar to the co-injection experiment (data not shown).

In the natural infection, the IFN-α response in patients was found to be low. The genomic analysis of the gene expression profile in HBV-infected chimpanzees showed nearly no IFN-α response during the acute phase and through the whole infection [45]. The absence of IFN-α may be a result of low induction of IFN-α/β system by HBV but partly of HBV-mediated inhibition of IFN-α/β signaling. Recently, Chen et al. demonstrated that HBV polymerase is able to block IFN-α signaling by inhibition of STAT1 phosphorylation by PKC-δ and importin-mediated nuclear translocation of STAT1/STAT2 complex [46]. These mechanisms were also active in the mouse model, explaining our observation that ISGs expression was reduced in the presence of HBV replication (Fig. 4). Nevertheless, IFN-α is able to finally clear HBV. For acute HCV infection, early IFN-α therapy is recommended. It is not clinically evaluated whether acute HBV infection could be treated with IFN-α.

An interesting finding in our study is the different susceptibility of HBV isolates to IFN-α. The clinical data suggested a significant role of HBV genetics for the success rate of IFN-α therapy. Using our established system, it will be possible to analyze the HBV genetic determinants for IFN-α susceptibility/resistance by HI pmIFN-α directly into mouse liver. Until now, the role of HBV variants in IFN-α therapy is not well understood.

In HBeAg positive patients treated with conventional IFN-α, a higher rate of HBsAg loss [12] and HBeAg clearance was observed for genotype A compared with non-A genotypes, not only as a result of IFN-α based therapy but also spontaneously [47]. However, we got an opposite result that HBV genotype B isolate was more sensitive to IFN-α treatment than HBV genotype A isolate. We speculated that this is probably associated with the presence of genotype-dependent mutations located within the basic core promoter (BCP) region of the HBV genome [48]–[49].

Viral genetic factors have been poorly investigated for their effect on the outcome of IFN-α therapy. G1896A mutation, converting a tryptophane (TGG) into a translational stop codon (TAG), has been reported to be associated with a bad prognosis and a poor response to IFN-α therapy. Mutations in the polymerase gene play an important role for resistance to nucleoside analogues, but do not seem to have an effect on the outcome of IFN-α therapy. As yet the relevance of mutations of the HBV core promoter region for responsiveness to IFN-α has not been evaluated.

BCP contains *cis*-acting elements that independently direct the transcription of pre-C RNA and pgRNA [50]. Double mutant A1762T/G1764A within BCP domain has decreased HBeAg expression and increased HBV replication in vitro [51]–[52]. The same result was observed in our study. Because HBeAg can inhibit HBV replication [53]–[54], the reduced expression of HBeAg could be a reason for increased viral replication. The increase of the viral replication is likely the reason why this double mutation often becomes the major quasispecies during chronic infection. Moreover, A1762T/G1764A is non-synonymous mutant and could affect the structure of the X protein, which functions as a transcriptional *trans*-activator of viral and cellular genes and is required for the establishment of hepadnaviral infection in animals [55]–[56]. It has been speculated that the immune reaction against infected hepatocytes may be increased as a result of a reduced HBeAg expression and that an enhanced viral replication may even show a cytopathic effect to infected liver. Nevertheless, the precise role of the A1762T/G1764A and the potential impact on viral replication and immune response must be elucidated in future.

## Supporting Information

Fig. S1 **The construction procedure for pAAV-HBV-B is shown.** pAAV-HBV-B was verified by restriction enzyme digestion with BamHI or HindIII, respectively. When pAAV-HBV-B was digested by *Bam*HI, two bands at the sizes of 3215 bp (full-length HBV fragment) and a fragment corresponding to the cloning vector were visible (A). The plasmid pAAV-HBV-B was digested by *Hin*dIII that cuts the only at a single site (B).
(TIF)
