# Supplementary figures and images for: Susceptibility of Different Hepatitis B Virus Isolates to Interferon-Alpha in a Mouse Model Based on Hydrodynamic Injection
Source: PLoS One. 2014 Mar 11;9(3):e90977. doi: 10.1371/journal.pone.0090977 (PMC3950299; doi:10.1371/journal.pone.0090977)

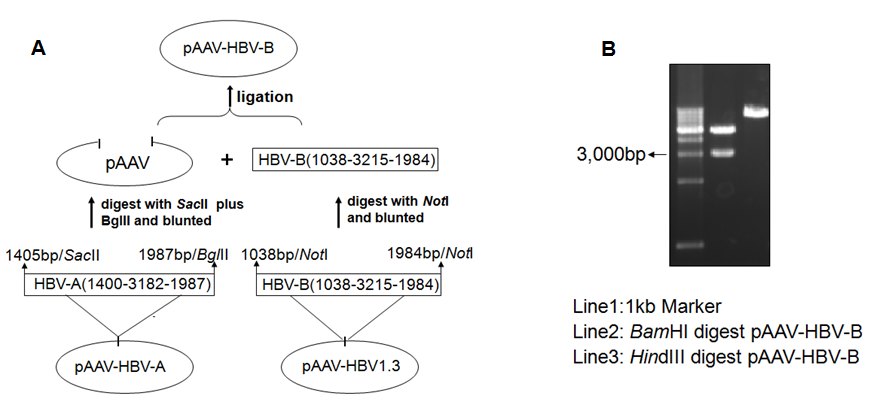

Supplement: Fig. S1 — The construction procedure for pAAV-HBV-B is shown. pAAV-HBV-B was verified by restriction enzyme digestion with BamHI or HindIII, respectively. When pAAV-HBV-B was digested by BamHI, two bands at the sizes of 3215 bp (full-length HBV fragment) and a fragment corresponding to the cloning vector were visible (A). The plasmid pAAV-HBV-B was digested by HindIII that cuts the only at a single site (B). (TIF) [file pone.0090977.s001.tif]
